# Paroxysmal nocturnal haemoglobinuria, diagnosis and haematological findings, first report from Iran, model for developing countries

**DOI:** 10.1002/jha2.410

**Published:** 2022-03-08

**Authors:** Mohammadali Jahangirpour, Amirali Vahedi, Hamed Baghdadi, Tahereh Madani, Ali Behvarmanesh, Mohammad Alidadi, Mohadese Hashem Boroojerdi, Saba Mohammaei, Peyvand Poopak, Amirhossein Poopak, Gelareh Khosravi Pour, Behzad Poopak

**Affiliations:** ^1^ Portiuncula University Hospital Ballinasloe Co. Galway Republic of Ireland; ^2^ Payvand Clinical and Specialty Laboratory Tehran Iran; ^3^ Department of Hematology Faculty of Medical Sciences Tarbiat Modares University Tehran Iran; ^4^ Department of Biochemistry and Centre de Recherche en Biologie Structurale McGill University Montreal Quebec Canada; ^5^ In Vitro Medical Device Department National Medical Device Directorate Food And Drug Administration Tehran Iran; ^6^ Department of Medical Biotechnology, Medical Faculty, Tarbiat Modares University Tehran Iran; ^7^ Tehran University of Medical Sciences Tehran Iran; ^8^ Students’ Scientific Research Centre Tehran University of Medical Sciences Tehran Iran; ^9^ Islamic Azad University Tehran Medical Sciences Branch Tehran Iran

**Keywords:** anaemia, PNH, thrombosis

## Abstract

Since paroxysmal nocturnal haemoglobinuria (PNH) was first described in 1881, the diagnosis and follow‐up patients diagnosed with the illness has remained an area of concern, with several different techniques of varying sensitivity having been described in the literature for both the diagnosis and monitoring treatment of the disease. PNH is a rare and life‐threatening disease that manifests symptoms of haemolytic anaemia. Hence, a quick and reliable technique for precise diagnosis would be crucial. PNH patients who have previously been diagnosed with aplastic anaemia or myelodysplastic syndrome carry small PNH clones and for more than a century traditional method with low sensitivity was used for such patients. In 2010, the International Clinical Cytometry Society described a highly sensitive method for detection and quantification of different types of PNH clones using multi‐colour flow cytometry. In this method, a three‐colour flow cytometer is essential to detect PNH affected cells amongst monocytes and granulocytes. This started a new era in the diagnosis of patients who carry small clones of PNH cells. Before this, flow cytometric analysis was used only for detection of PNH cells amongst erythrocytes. By using flow cytometry instruments with more light sources, the sensitivity of detection and quantification of PNH clones would be augmented. However, standardisation and crosstalk compensation would be the most concerning issue. For the first time in Iran, we set up and standardised multi‐colour flow cytometry technique to detect PNH cells in erythrocytes and leukocytes at Payvand medical laboratory.

## 1 INTRODUCTION

Paroxysmal nocturnal haemoglobinuria (PNH) is an acquired and rare clonal blood disorder of haematopoietic stem cells [1]. It has been shown that there is a partial or absolute deficiency in the biosynthesis of the glycophosphatidylinositol (GPI)‐anchored proteins in various cell lineages (lymphoid and myeloid) in PNH patients, caused by mutations in the phosphatidylinositol glycan complementation class A (*PIGA*) gene on the X‐chromosome [2]. CD14, CD16, CD24, CD55 and CD59 are common GPI‐anchored proteins on the surface of granulocytes, monocytes and erythrocytes [3, 4]. Many of these proteins have key regulatory roles in the complement system such as CD55 (decay accelerating factor) and CD59 (membrane inhibitor of reactive lysis); a lack of these two proteins specifically results in an increased vulnerability of the red blood cells (RBCs) to haemolysis by the complement system [3]. Intravascular haemolysis leads to haemoglobinuria and presence of haemosiderin [5] in urine and results in urine discolouration to a red or brown colour in night‐time and early morning urine samples [5]. Elevated serum lactate dehydrogenase (LDH) and bilirubin as well as increased reticulocyte count with a low serum haptoglobin are laboratory hallmarks of intravascular haemolysis [6]. Thrombosis, especially at uncommon sites, is another finding in PNH patients [7] and thrombosis intensity has been correlated with the size of the PNH clone [3]. Fatigue, dyspnoea, abdominal pain and discolouration of urine are the main complaints of almost all patients [8]. However, these symptoms are not specific for PNH and can be seen in many other intravascular haemolytic disorders.

PNH is a rare disease with an incidence of 1–1.5 cases per million and worldwide studies show both men and women are affected by PNH equally. However, some studies have showed slightly higher rate in women rather than men [9]. It has a higher prevalence in Asian countries than western countries based on the data obtained from the International PNH registry [1, 10]. PNH may be associated with aplastic anaemia (AA), myelodysplastic syndromes (MDS) or acute myeloid leukaemia (AML) [11] and many cases develop in patients who were previously diagnosed with AA or MDS. In recent years, the median survival rate has increased to approximately 10–15 years, but this varies greatly between patients [7, 12].

Thrombosis is the main cause of death in all patients and rate of thrombosis in patients with a large PNH clone is more significant than other PNH patients [7]. Historically diagnosis of PNH was based on finding the sensitivity of RBCs to complement mediated haemolysis by use of a sucrose lysis test and confirmation by Ham's test [3, 13]. Later, another test was introduced known as complement lysis sensitivity test, which measures the percentage of haemolysis in a spectrum concentrations of complement [14]. This test was more specific and by use of it, different types/clones of PNH were discovered. Type II PNH patients show a population of RBCs with intermediate sensitivity to complement and type III PNH patients have the most abnormal/sensitive RBCs, whereas type I are classed as normal erythrocytes [15]. But this test is very difficult to standardise and cannot detect small populations of abnormal cells.

 All these tests are based on haemolysis of RBCs in different conditions. Today, it is well‐understood that not only RBCs but also granulocytes and monocytes are affected by PNH [16] and the diagnosis of PNH, particularly in patients with a small population of abnormal cells, needs a highly sensitive technique. In 2010, the International Clinical Cytometry Society (ICCS), recommended a highly sensitive method for the detection of PNH cells using multi‐parameter flow cytometry [16]. Using this method, the sensitivity of detecting PNH cells has increased 100 times (from 1% by old techniques to 0.01% using multi‐parameter flow cytometry). Detection and monitoring PNH clones, particularly in AA and MDS patients is crucial, hence a highly sensitive method that is able to detect a small clone of PNH cells plays a pivotal role.

According to ICCS guidelines, we standardised assays for the detection of PNH cells in both RBC and white blood cell (WBC) lineages. Selection of RBCs through Glycophorin A (CD235a) and detecting CD59 and CD55 on their surface is the best method to recognise and quantify normal RBCs (type I) from type II and type III PNH cells. However, CD59 and CD55 are not the preferred markers to detect PNH affected leukocytes amongst monocytes and granulocytes [17]. Accumulated data have shown fluorescent‐labelled aerolysin (FLAER) is the best available reagent to detect PNH clones on leukocytes. Proaerolysin is a protoxin of aerolysin and binds to GPI anchor on the surface of leukocytes. FLAER cannot be used to detect PNH clone on RBCs.

In the current study, we have reviewed and reported the clinical manifestations, haematologic findings and flow cytometry analysis of our PNH cases at the Payvand medical laboratory between 2014 and 2019 that had been diagnosed using three‐ and four‐colour flow cytometry.

## 2 MATERIALS AND METHODS

### 2.1 Samples

A total number of 671 samples collected from patients have been referred for PNH investigation at the Payvand medical laboratory between 2014 and 2019 and all signed the corresponding consent.

Ethylenediaminetetraacetic acid (EDTA) anticoagulated fresh blood samples were collected from patients who were referred to the laboratory for PNH assessment. Full blood count (FBC) tests were performed using the Mindray's BC‐6800 and the ADVIA^®^ 2120i Haematology Systems.

### 2.2 Monoclonal antibodies

CD235a Fluorescein isothiocyanate (FITC) (clone10F7MN, eBiosciences), CD59 Phycoerythrin (PE) (clone H19, BD Biosciences) and CD55 PE (clone IA10, BD Biosciences) were used for RBC immunophenotyping. FLAER (Alexa 488, Cedarlane), CD45 Allophycocyanin (APC) (clone D3/9, BD Biosciences), CD15 PerCP‐Cy5.5 (HI98, BD) and CD24 PE (SN3, Life technologies) were used for granulocyte immunophenotyping. FLAER (Alexa 488, Cedarlane), CD45 APC (D3/9, BD Biosciences), CD64 PE‐Cy5 (22, Beckman Coulter) and CD14 PE (TuK4, Life Technologies) were used for monocyte immunophenotyping.

### 2.3 Methods

In the current study, all the RBC/WBC staining procedures and gating strategies were carried out based on the practical guideline provided by Sutherland et al. According to our laboratory's protocol, the optimal titration for CD235a FITC is 2 µl; 5 µl for CD59 PE; 5 µl for CD55 PE; 2 µl for FLAER; 2 µl for CD45 APC; 2 µl for CD15 PerCP‐Cy5.5; 2 µl for CD24 PE; 2 µl for CD64 PE‐Cy5; and 2 µl for CD14 PE.

### 2.4 Instruments and software

PNH clone assessment was performed using a three‐colour assay on Sysmex Partec CyFlow Space instrument and analysed by FlowMax software. Later, due to the improvement of the instrument in our laboratory, we started to use the four‐colour assay on BD FACSLyric flow cytometer and BD FACSuite 1.2.1 software.

### 2.5 Statistical analysis

All data were analysed and were examined for validity and reliability using the GraphPad Prism version 8.3.1.

### 2.6 Verification and validation

For both RBC and WBC assays, samples of normal patients were used to verify that the PNH gate did not have any background events, and to compare the abnormal population with type I RBCs which are normal cells. For determining PNH clones ‘fluorescence minus two’ staining was performed. In this method, we used gating markers including CD235‐Side scatter, CD15‐CD45 and CD64‐CD45, but not GPI‐linked markers. This population will appear as GPI negative population, so we can compare them with PNH clones, particularly the highly abnormal type III clones.

## 3 RESULTS

Out of 671 referred patients, 81 (12.1%) of them with a mean age of 38 years (range 11–79 years) were diagnosed with PNH and 49 (60.5%) out of 81 patients were between 25 and 45 years. Males constitute 53.1% (43 out of 81) of the patients and females constitute 46.9% (38 out of 81) of those diagnosed.

### 3.1 Clinical manifestation

Out of all 81 patients only 59 of them had available clinical history and of those, 38 patients had a history of anaemia. Some common symptoms described by this cohort include fatigue in eight patients, jaundice and pale skin in five patients, haematuria in four patients, abdominal pain in four patients and bruises in five patients. Furthermore, 13 patients have previously presented with AA.

### 3.2 Haematological features

In this study, a total of 51.8% of our patients showed leukopaenia with an average WBC count of 2.18 × 10^3^/µl, with a range between 0.48 and 3.92 × 10^3^/µl. Leukopaenia was defined as having a WBC less than 4 × 10^3^/µl according to the protocols at the Payvand medical laboratory. Also, 74 (91.4%) out of 81 patients showed anaemia with an average haemoglobin of 8.9 g/dl (range: 7–11.8 g/dl) in women and 9.7 g/dl (range: 7.1–12.7 g/dl) in men. Anaemia was defined as a having a haemoglobin concentration less than 12 and 13 g/dl for women and men, respectively. Amongst these 74 patients with anaemia, seven women (9.5%) with an average haemoglobin of 6.1 g/dl, and eight men (10.8%) with an average haemoglobin of 6.1 g/dl were diagnosed with severe anaemia (18.5% of total patients) which was defined by a haemoglobin below 7 g/dl. In addition, 60 individuals (74.1%) were diagnosed with thrombocytopaenia with an average platelet count of 51.3 × 10^3^/µl, overall. Thrombocytopaenia was defined as having a platelet count less than 145 × 10^3^/µl. Table 1 demonstrates the separate and simultaneous deficiency of the abovementioned blood parameters in more detail.

**TABLE 1:** Detailed information of white blood cell (WBC), haemoglobin (Hb) and platelet (PLT) deficiency in paroxysmal nocturnal haemoglobinuria (PNH) patients

	Low WBC	Low Hb	Low PLT	Low WBC and Hb	Low WBC and PLT	Low Hb and PLT	Low WBC, Hb and PLT	Normal
Male	0	7	3	2	1	11	19	0
Female	0	10	2	1	0	5	19	1
Total	0	17	5	3	1	16	38	1
Percentage	0%	20.99%	6.17%	3.70%	1.23%	19.75%	46.91%	1.23%

Furthermore, 51.9% of patients suffered from neutropaenia including 22 males (with the average neutrophil count of 0.81 × 10^3^/µl) and 20 females (with the average of 0.83 × 10^3^/µl). According to our standard guidelines at the Payvand medical laboratory, the lower threshold of neutrophil count was set to 1.8 × 10^3^/µl in males and 2 × 10^3^/µl in females. Also, 42% of the patients were diagnosed with lymphopaenia including 26 males and eight females with the average lymphocyte count of 1.01 × 10^3^ and 0.43 × 10^3^/µl, respectively. The lower threshold for lymphocyte count was defined as 1.5 × 10^3^ and 0.8 × 10^3^/µl in males and females, respectively. Moreover, 29.9% presented with monocytopaenia consist of 13 males (average monocyte count of 0.06 × 10^3^/µl) and 10 females (average 0.05 × 10^3^/µl). The lower threshold for monocyte count in both genders was set to 0.12 × 10^3^/µl.

### 3.3 PNH clone assessment using flow cytometry

Results of the PNH clone assessment show that the total PNH clone size on RBCs ranges from 0.34% to 87.8% with an average of 29.58%. The average PNH clone size on monocytes is 58.85% (range: 1.59%–99%). Analysis of the PNH clone size on granulocytes reveals that 62.96% of the PNH clones are above 50% (large PNH clones), 17.28% are between 10% and 50% and 19.75% of the clones are less than 10%. PNH clone size on granulocytes ranges between 0.07% and 98.44% with an average of 58.94%.

**FIGURE 1:**
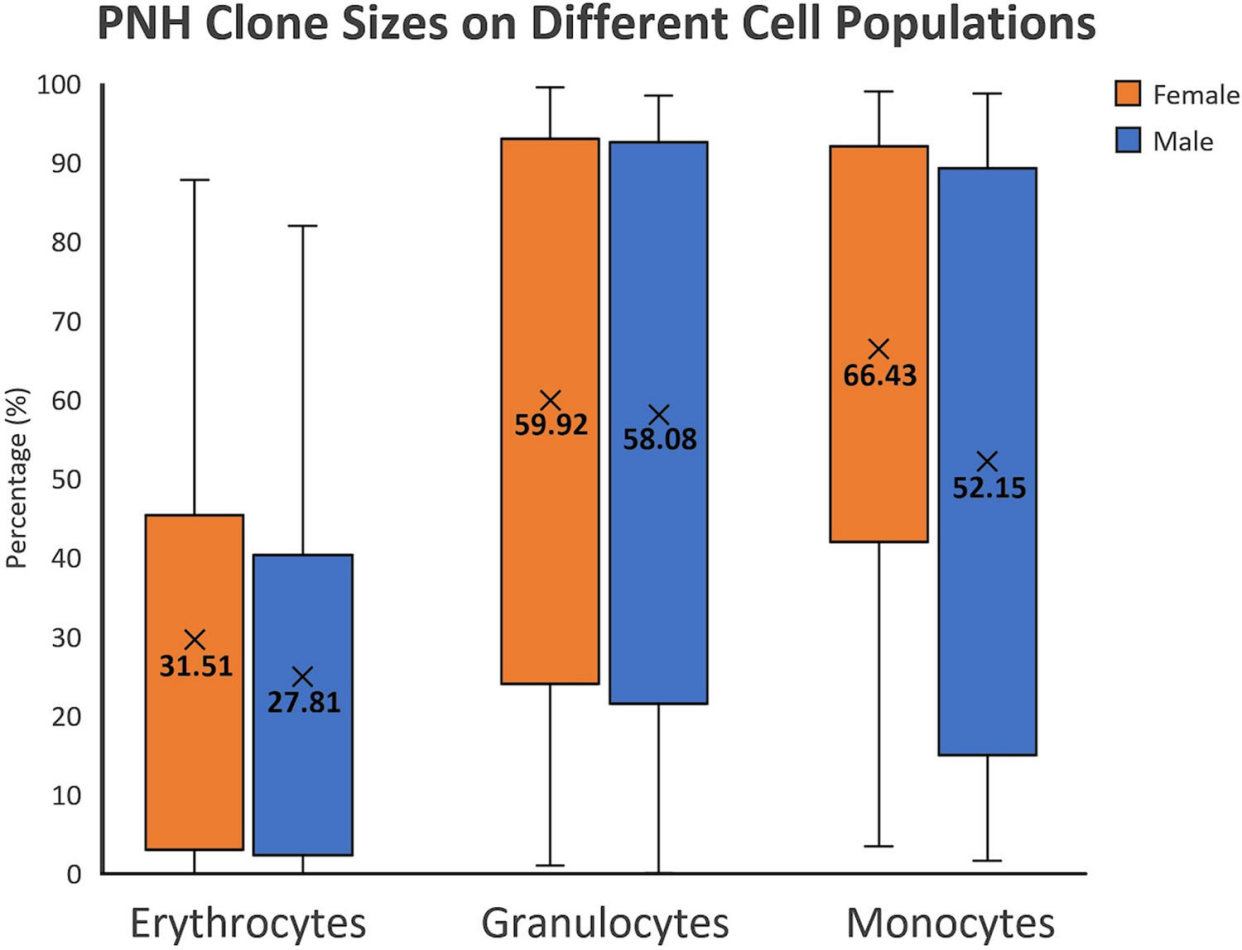
The average paroxysmal nocturnal haemoglobinuria (PNH) clone sizes on red blood cell (RBC), granulocyte and monocyte in both genders

Figure 1 compares the average percentage of PNH clones on each cell lineages between males and females. It initially appears that the percentage of clones are higher in females, but a Mann–Whitney *U*‐test shows that this difference is not statistically significant with the *p*‐value of 0.5131, 0.5737 and 0.1127 for RBC, granulocytes and monocytes, respectively.

**FIGURE 2:**
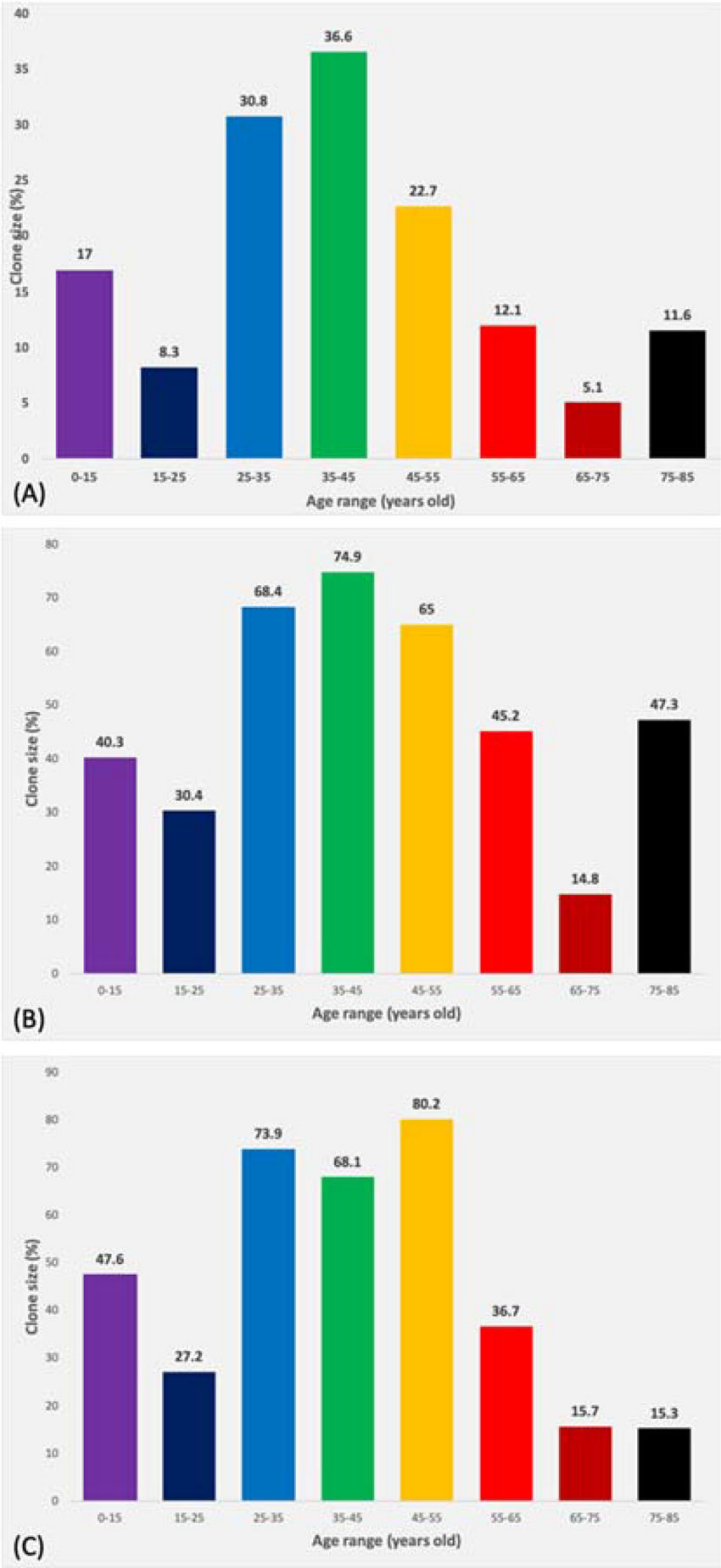
The bar charts compare the average paroxysmal nocturnal haemoglobinuria (PNH) clone sizes (in percentage) on (A) red blood cells (RBCs), (B) granulocytes and (C) monocytes in different age groups

When the average PNH clone size on each cell line is analysed across various age groups, the 25–45 years old age range shows higher percentages of a PNH clones on average across the three different cell lines, followed by the 45–55 years old age range (Figure 2).

The average PNH clone size in RBCs is 36.58% and 30.81% in the 35–45 and 25–35 years old age ranges, respectively; followed by an average PNH clone size of 22.71% in the 45–55 years old age group. The PNH clone percentage on granulocytes follows the same trend as the RBC clone size data, with 74.88%, 68.4% and 65.02% in 35–45, 25–35 and 45–55 years old age ranges, respectively. However, the average PNH clone size on monocytes shows the 45–55 years old cohort at the top with 80.2% PNH clone average, followed by 73.91% average clone size in 25–35 years old range, whilst the 45–55 years old age range stands in third place with an average PNH clone size of 68.05%.

Results of the Mann–Whitney test indicate that the differences between the clone size percentages are significantly different between the 25–45 years old age range and other age ranges. The *p*‐value of the PNH clone size in Mann–Whitney test was <0.0001 for RBCs, 0.0001 for granulocytes and 0.0002 for monocytes.

A comparison between the PNH clone sizes in 13 patients who had been diagnosed with AA versus other non‐AA patients, shows that the size of the clones on different cell lineages are not significantly different in these two groups according to the results of the Mann–Whitney test which calculates the *p*‐value > 0.05.

## 4 DISCUSSION

PNH is an acquired blood disorder that occurs due to a mutation in the *PIG‐A* gene and presents with various clinical signs and symptoms [18]. The ICCS suggests flow cytometry as a highly sensitive and widely accepted technique for the diagnosis of abnormal PNH clones. The current study is the first ever comprehensive PNH case report from Iran, which was carried out from 2014 to 2019 in a single centre and attempts to provide a clear picture of the burden of PNH in Iran and compares it to those reported in other regions.

The results of this study suggest a slight male predominance with 53.1% of the total patients diagnosed with PNH being male and this follows the same trend that has been reported by other studies in Asia. However, it has been shown in western countries that females suffer from this disease more than males [1, 18]. The distribution of the PNH patients in our laboratory shows that 69.14% of the patients are between 25 and 55 years old which is consistent with the data from other studies. Socié et al. reported the 30–59 years old age range as the most prominent age group by analysing the PNH registry data [10]. Furthermore, young adults with an average age of 38 years have been reported as the most commonly affected age group in Asian countries according to Yu et al. [18].

**FIGURE 3:**
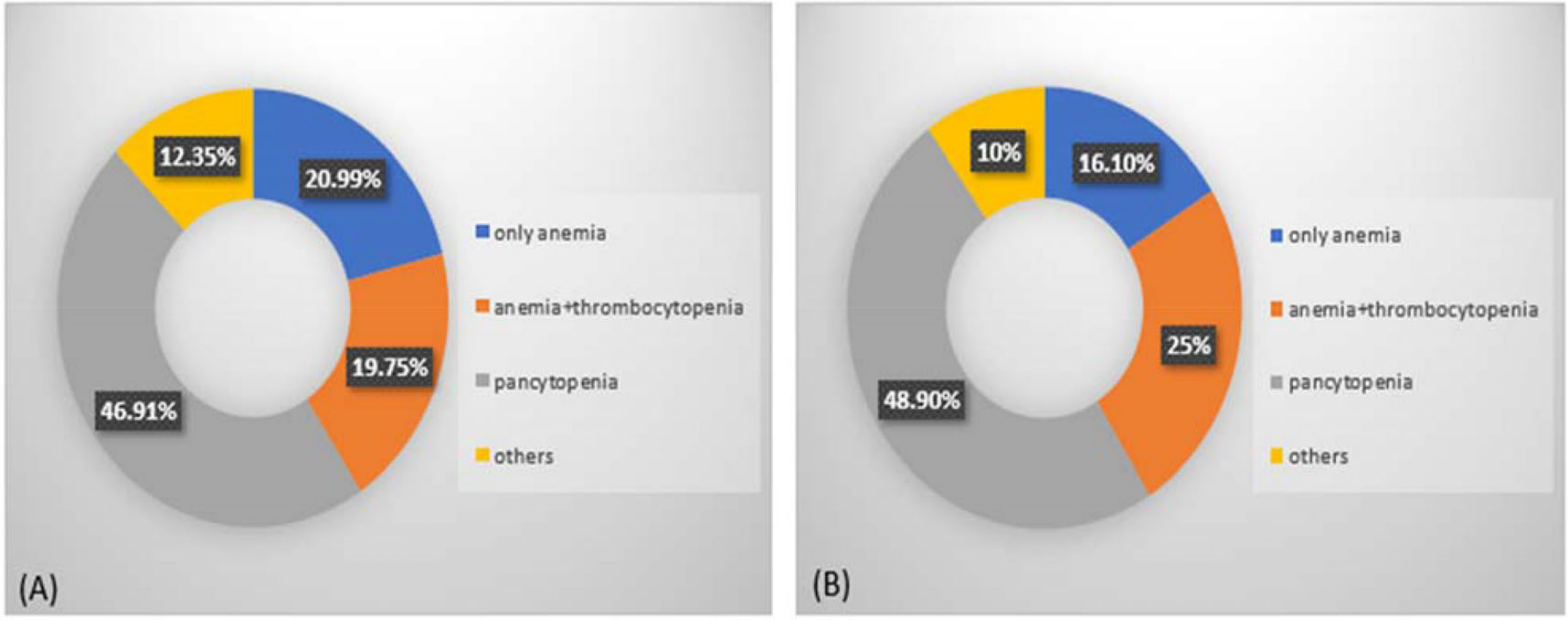
Compares the blood parameters data between (A) observations in this study and (B) the results obtained from a former study in Asia. The ‘others’ section in yellow colour stands for the percentage of patients with other types of deficiencies including low white blood cell (WBC), low platelet, bicytopaenia other than low haemoglobin and platelet, and normal cases

Looking at the FBC data from those diagnosed with PNH (haemoglobin, WBC and platelets) indicate that nearly half of our patients (46.9%) suffered from pancytopaenia whilst 24.7% had a deficiency in two of the examined parameters simultaneously; this trend is almost the same as the one that has been reported by Yu et al. [18] in other Asian countries (Figure 3).

In agreement with former studies, results of the current report show that the PNH clone sizes on RBCs are significantly smaller than clones on WBCs with a *p*‐value <0.0001 using a Mann–Whitney test. This difference may be due to previous blood transfusions or the increased rate of haemolysis within this cohort of patients [19]. There's also a significant correlation between PNH clone sizes on different cell lineages with a Spearman *r* of 0.7316 for PNH clones on granulocytes and monocytes, and a Spearman *r* of 0.7813 for PNH clones on RBCs and granulocytes. Correlation between the clone sizes on RBCs and monocytes is not as strong as the others (Spearman *r* = 0.6865).

The comparison between the PNH clone sizes on different cell lineages in 13 of our patients who had been diagnosed with AA versus other non‐AA patients suggests that the average size of PNH clones are smaller in AA patients, which is consistent with the results of the previous reports [10, 20]; however, this difference is not statistically significant with a *p*‐value of 0.2176, 0.4128 and 0.5996 in Mann–Whitney test for PNH clones on RBC, granulocytes and monocytes, respectively.

When the PNH clone size was examined across different age groups in each of the cell lineages, it was found that on average the clone size were largest across all cell lineages in the 25–45 years age group. This finding would be suggestive that this age group presented on average with more severe disease, according to Gupta et al. [20].

Finally, authors want to declare that due to incomplete clinical history and a limited number of PNH patients the data might be biased and only represents a portion of the Iranian patients. Therefore, a larger, multi‐central dataset analysis of PNH patients with a more comprehensive clinical history is recommended for future studies and metanalysis.

## CONFLICT OF INTEREST

None of authors declared any conflict of interest.
